# Dr. Francis Hartman Markoe

**Published:** 1907-10

**Authors:** 


Dr. Francis Hartman Markoe, of New York, son of the late Dr. Thomas M. Markoe, died at his home September 13, 1907, aged 51 years. He was a graduate of the College of Physicians and Surgeons, 1879, and served on the surgical house staff of the New York Hospital, where he afterward became attending surgeon. He also taught surgery in the College of Physicians and Surgeons and became one of the attending surgeons at St. Luke's Hospital. The New York Medical Journal very justly says: "Not within our memory has death deprived the New York profession of a more highly esteemed or more valuable member."

